# The MYC*/miR-17-92* axis in lymphoproliferative disorders: A common pathway with therapeutic potential

**DOI:** 10.18632/oncotarget.4574

**Published:** 2015-07-21

**Authors:** Michele Dal Bo, Riccardo Bomben, Luis Hernández, Valter Gattei

**Affiliations:** ^1^ Clinical and Experimental Onco-Hematology Unit, Centro di Riferimento Oncologico, I.R.C.C.S., Aviano PN, Italy; ^2^ Department of Pathology, Hospital Clinic, Institut d'Investigacions Biomèdiques August Pi i Sunyer (IDIBAPS), University of Barcelona, Barcelona, Spain

**Keywords:** miR-17-92 cluster, lymphoproliferative disorders, anti-miRNA based target therapy

## Abstract

MicroRNAs (miRNAs) represent a class of small non-coding single-stranded RNA molecules acting as master regulators of gene expression post transcriptionally by inhibiting the translation or inducing the degradation of target messenger RNAs (mRNAs). In particular, the *miR-17-92* cluster is widely expressed in many different cell types and is essential for many developmental and pathogenic processes. As a strong oncogene, *miR-17-92* can regulate multiple cellular processes that favor malignant transformation, promoting cell survival, rapid cell proliferation, and increased angiogenesis. The *miR-17-92* cluster has been reported to be involved in hematopoietic malignancies including diffuse large B-cell lymphoma, mantle cell lymphoma, Burkitt's lymphoma, and chronic lymphocytic leukemia. Given the multiple and potent effects on cellular proliferation and apoptosis exerted by the *miR-17-92* cluster, miRNAs belonging to the cluster surely represent attractive targets for cancer therapy also in the context of lymphoproliferative disorders. In the present review, we focus on the role of the *miR-17-92* cluster in lymphoproliferative disorders, including diagnostic/prognostic implications, and on the potential applications of anti-miRNAs based therapies targeting miRNAs belonging to the cluster.

## INTRODUCTION

MicroRNAs (miRNAs) represent a class of small non-coding single-stranded RNA molecules of 17–27-nucleotides in length that act as master regulators of gene expression post-transcriptionally by inhibiting the translation or inducing the degradation of target messenger RNAs (mRNAs) with partially complementary sites in the 3′-untranslated regions [1, 2]. Given these multiple roles, miRNAs aberrant expression and dysregulation often results in human diseases and cancer [3–9]. The first miRNAs related with cancer were *miR-15a/miR-16-1* whose cluster is located in the chromosome region 13q, commonly deleted in chronic lymphocytic leukemia (CLL) [10]. The deletion was proposed as one of the primary genetic event in CLL, due to a decreased expression of the *miR-15a/miR-16-1* targeting the *BCL2* gene [10]. Other evidence of the association between miRNA dysregulation and cancer came from studies revealing that many miRNAs are effectively located in genomic regions frequently involved in chromosomal alterations, including breakpoint deletions or amplifications related to cancer [11, 12]. Overall considered, these studies indicate that the contribute of miRNAs to cancer pathogenesis is dependent of two opposite functions: either they can act as tumor suppressors, as in the case of the *miR-15a/miR-16-1* cluster in CLL, or they can act as oncogenes, as it has been proposed for the members of the *miR-17-92* cluster that is the topic of the present review [13].

In this review we first describe the *miR-17-92* cluster locus along with the main mechanism(s) of expression regulation and the major molecular interactions of the *miR-17-92* cluster in normal and neoplastic B cells. Then, we review the main clinical and pathogenetic implications of *miR-17-92* cluster expression in lymphoproliferative disorders and the potential applications of anti-miRNAs based therapies targeting miRNAs belonging to the cluster.

### The *miR-17-92* chromosomal locus

**Figure 1:**
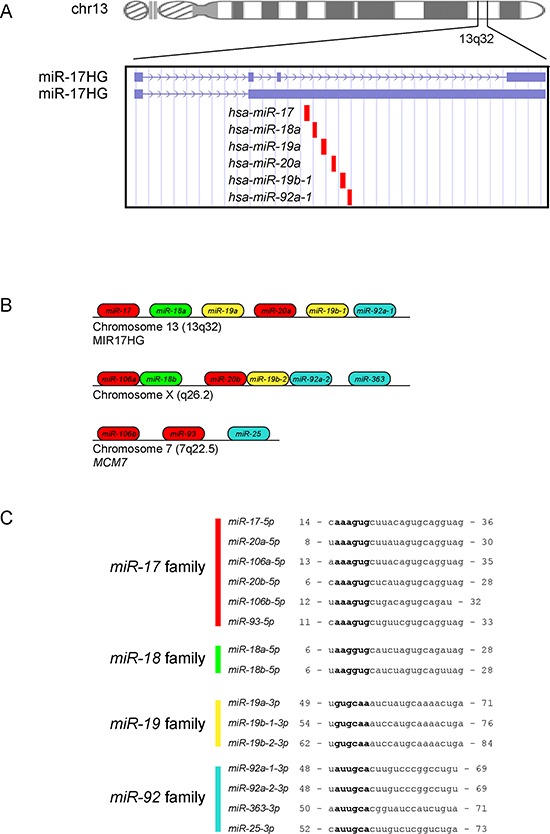
The *miR-17-92* cluster **A.** Genomic localization of the *miR-17-92* cluster (*MIR17HG*). The two transcripts of the gene are reported in blue. The miRNAs belonging to the cluster are reported in red. The panel of the figure was created modifying the output of the UCSC genome browser. **B.** Structure of the *miR-17-92* cluster (*MIR17HG*, located on chromosome 13) and of the two paralogs (located on chromosomes X and 7). Both paralogs contains homologous miRNAs to a subset of *miR-17-92* components. **C.** miRNA families of the *miR-17-92* cluster. Four separate miRNA families according to miRNA seed sequences have been defined: the *miR-17*, *miR-18*, *miR-19* and *miR-92* families. *miR-17*, *miR-18* and *miR-19* families are composed also by miRNAs belonging to the paralogs. Seed sequences are reported in bold.

The *miR-17-92* polycistronic miRNA cluster is located in a region of 800 bp in the non-protein-coding gene C13orf25 at 13q31.3 (Figure 1A) [14]. The precursor transcript derived from the *miR-17-92* gene (a.k.a. *MIR17HG*) contains six tandem-loop hairpin structures that ultimately yield the six mature miRNAs *miR-17*, *miR-18a*, *miR-19a*, *miR-20a*, *miR-19b-1* and *miR-92a-1* [14]. Moreover, complementary miRNAs derived from the opposite strands of each *miR-17-92* pre-miRNA have been identified. The biological importance of the *miR-17-92* cluster is also underlined by the presence of paralogs on chromosome X and chromosome 7, the *miR-106a-363* cluster and the *miR-106b-25* cluster, respectively, that both contains homologous miRNAs to a subset of *miR-17-92* components (Figure 1B). Three separate miRNA families according to miRNA seed sequences have been defined: the *miR-17* (that includes *miR-17*, *miR-20a* and *miR-18a*), *miR-19* (that includes *miR-19a* and *miR-19b-1*), and *miR-92* families (Figure 1C). Both *miR-17* and *miR-19* families are composed also by miRNAs belonging to the paralogs (Figure 1C). All these miRNAs derive from an unique gene that, during the early evolution of vertebrates, underwent a series of different dysregulations, such as duplications, mutations and losses [15].

The six members of the *miR-17-92* cluster can act independently and/or coordinately to target various mRNA, according to the degree of binding affinity and the seed sequences of the various members of the cluster (Figure 1C). As a strong oncogene, *miR-17-92* regulates multiple cellular processes that favor malignant transformation promoting cell survival, rapid cell proliferation, and increased angiogenesis [16–19]. Given the oncogenic role of the *miR-17-92* cluster, the primary transcript for these miRNAs was named ‘*OncomiR-1*' [17].

The 13q31.3 human genomic locus undergoes amplification in several types of lymphoproliferative disorders and solid tumors [20, 21], and, consistently, aberrant overexpression of the *miR-17-92* cluster in the absence of amplification is also frequently observed in some tumors. The *miR-17-92* cluster has been reported to be involved in hematopoietic malignancies including diffuse large B-cell lymphoma (DLBCL), mantle cell lymphoma (MCL), Burkitt's lymphoma (BL), and CLL (Table 1) [14, 21–26]. In general, a significant over expression of pri- *miR-17-92* has been observed in 65% of B-cell lymphoma patients [17].

**Table 1:** *miR-17-92* cluster overexpression in lymphoproliferative disorders

Disease	*miR-17-92* dysregulation	Genomic aberrations	References
DLBCL	overexpression	13q31.3 amplification; *MYC* aberration in complex karyotypes	[17]; [26]; [57]; [58]; [59]; [61]; [62]; [64]; [65]; [56]
MCL	overexpression	13q31.3 amplification	[24]
BL	overexpression	13q31.3 amplification; *MYC* translocation	[77]; [78]; [79]; [80];[81]; [82]
CLL	overexpression upon microenvironmental stimuli compared to the unstimulated counterpart	absence of associated genomic aberrations	[22]; [94]

Abbreviations: DLBCL, diffuse large B cell lymphoma; MCL, mantle cell lymphoma; BL, Burkitt's lymphoma; CLL, chronic lymphocytic leukemia.

### Expression regulation of the *miR-17-92* cluster

**Figure 2:**
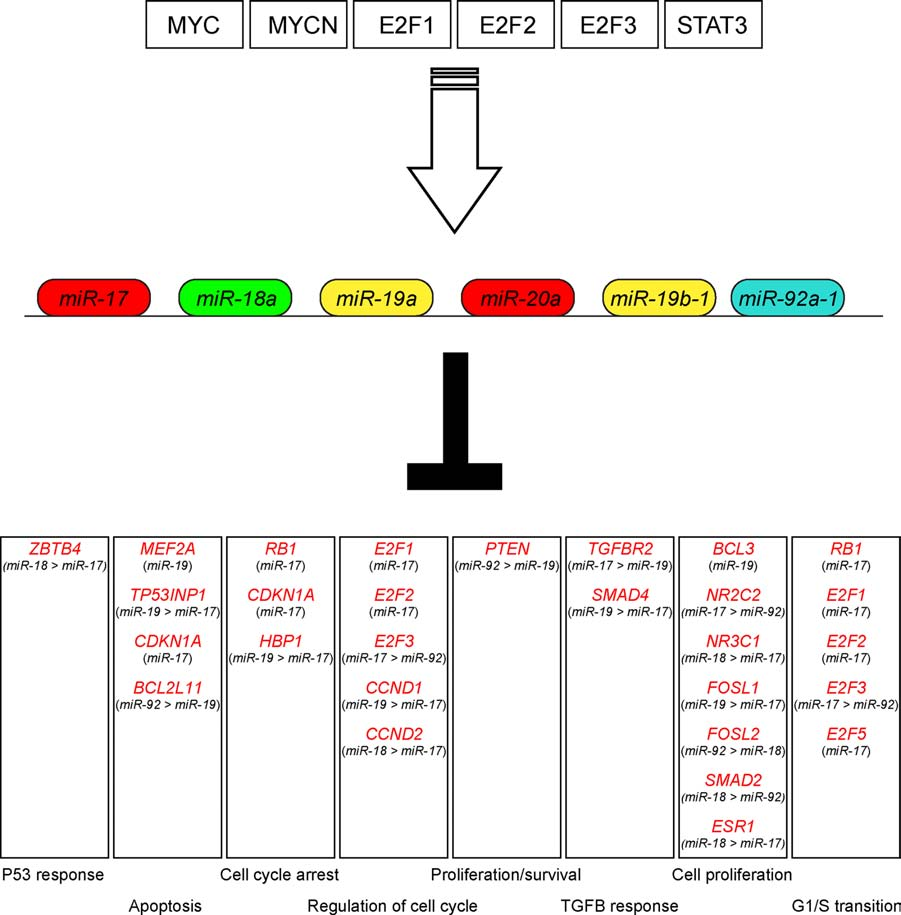
Major interactions of the *miR-17-92* cluster A number of oncogenic transcription factors positively (white arrow) regulate the expression of miRNAs from the *miR-17-92* cluster (upper part of the figure). miRNA belonging to the *miR-17-92* cluster target specific genes involved in different biological processes. The miRNA families (as indicated in Figure 1C) with the highest scores for each specific target gene are reported according to the algorithms Target Scan Human (http://www.targetscan.org) and http://Microrna.org; Targets and Expression (http://www.microrna.org).

A number of oncogenic transcription factors (TFs) regulate the expression of *miR-17-92* cluster thus influencing its oncogenic activity (Figure 2) [27, 28]. In particular, MYC, the first identified transcription regulator of *miR-17-92*, activates *miR-17-92* expression by directly binding to its genomic locus [17, 18, 29]. Similarly, *miR-17-92* can be also upregulated by MYCN, as demonstrated in neuroblastoma cells [30, 31]. In addition to MYC, TFs belonging to the E2F family, e.g. E2F1, E2F2, E2F3, are other potent inducers of the *miR-17-92* cluster [32]. The E2F TFs are essential for cell cycle progression; in fact, they activate a large number of S phase genes, including thymidine kinase, DNA polymerase, Cyclin A and Cyclin E. Thus, cycling cells are likely to have elevated levels of *miR-17-92* due to periodic burst of E2F activity during S phase, while quiescent cells may have reduced *miR-17-92* levels. Other TFs that directly activate the transcription of *miR-17-92* are two TFs belonging to the ETS family, SPI-1 and FLI-1, as demonstrated in the murine Friend's leukemia model [33]. Finally, STAT3 can also regulate the expression of the *miR-17-92* cluster at transcriptional level [34]. Notably, *miR-17-92* can be also transcriptionally repressed by tumor suppressors, e.g. TP53 in hypoxia-treated cells, or via histone modification [35–37].

### Major molecular interactions of the *miR-17-92* cluster

The major functional evidence of the oncogenic activity of *miR-17-92* comes from several studies employing B-cell lymphoma *in-vivo* mouse models. In a first model, in which *MYC* is driven by the immunoglobulin heavy chain enhancer (Eμ) as a transgene, the enforced expression of a truncated *miR-17-92* lacking the *miR-92a-1*, e.g. *miR-17-19b*, collaborated with the *MYC* to accelerate lymphomagenesis. Of note, the overexpression of *miR-17-19b* not only promotes the oncogenesis of MYC expressing B cells, but also alters the cell fate of transformed B cells. In fact, while the majority of B cell lymphomas derived from the Eμ-*MYC* transgene have a mature B-cell phenotype, B-cell lymphomas resulting from the collaboration between *miR-17-19b* and *MYC* are mostly derived from precursor cells [17]. In this context, a key oncogenic role is conducted by *miR-19a* and *miR-19b-1* as indicated by the fact that mutations of both *miR-19* miRNAs nearly abolish the oncogenic cooperation between *MYC* and *miR-17-92* [38–40]. On the other hand, in Eμ-*MYC* chimeric mice, it was demonstrated that this oncogenic cooperation is significant stronger when *miR-92a-1* is deleted within the oncomir, as well as when the seed sequence of the *miR-92a-1* is mutated [41]. Moreover, mutations of *miR-20a* or of *miR-17* did not affect oncogenesis in the Eμ-*MYC* model [41].

Regarding the oncogenic cooperation between *MYC* and *miR-17-92* [42], it has been demonstrated that *MYC* directly suppresses through *miR-17-92* the expression of the chromatin regulatory genes *SIN3B*, *HBP1*, *SUV420* and *BTG1* and the proapoptotic gene *BCL2L11* (a.k.a. *BIM*), this suppression contributing to maintain survival, autonomous proliferation, and self-renewal.

Moreover, according to another study [43], mice with exogenously induced *miR-17-92* overexpression in lymphocytes developed an aggressive lymphoproliferative disorder associated with autoimmunity followed by a premature death. This study also reported that, in the transgenic lymphocytes, the *miR-17-92* cluster down-regulated *PTEN* and *BCL2L11*, thus enhancing proliferation and survival [43]. This observation was confirmed by other studies employing B cell specific transgenic mouse models, in which *miR-17-92* overexpression induced B cell malignancies such as splenic B cell lymphomas or B cell leukemia/lymphomas [44, 45]. Seventeen genes, predicted to be targets of the *miR-17-92* cluster, have been found consistently downregulated in Eμ *miR-17-92* cells [45]. In transgenic mice specifically overexpressing *miR-17-92* in B cells [29], the *miR-17-92* cluster drives lymphomagenesis by suppressing the expression of multiple inhibitors of the PI3K and NFkB pathways and by inhibiting the mitochondrial apoptosis pathway [29]. In the same study, *miR-17-92*-driven lymphoma cells showed constitutive activation of the PI3K and NFkB pathways, and chemical inhibition of these pathways was useful to treat these lymphomas [29]. Moreover, as described in mice overexpressing *miR-17-92* in hematopoietic lineages [46], the expression of the *miR-17-92* cluster in a limited number of hematopoietic cells is sufficient to develop B cell malignancies, further highlighting the ability of *miR-17-92* to act as driver of tumorigenesis. On the other hand, the role of the *miR-17-92* cluster in the B cell development is also defined by the fact that a deficiency of *miR-17-92* impairs this process, particularly at the pro-B to pre-B transition stage, due to enhanced apoptosis occurring in the pro-B cells during both fetal and adult B cell development [19].

Among the miRNAs belonging to the *miR-17-92* cluster, *miR-17* and *miR-20a* are those that target the largest number of genes. In particular, these miRNAs are able to control the expression of genes with antagonizing functions, e.g. promoting or suppressing cell cycle progression [47], thus reflecting the complex and partially conflicting effects of miRNAs in tumor growth. In this context, the *E2F* TFs, direct targets of *MYC* [48], are among the genes down-regulated by *miR-17* and *miR-20a*. Thus, a tightly controlled proliferative loop is represented by *MYC* that simultaneously activates the *E2F* TFs and limits their translation by a miRNA-based mechanism [18]. In this process, a role can be exerted by RB1 and the other hypophosphorylated retinoblastoma proteins that inhibit *E2F* TFs and, in turn, are repressed by *miR-17* and *miR-20a* [49, 50]. *CCDN1* (a.k.a. Cyclin D1) is also regulated by *miR-17* and *miR-20a*. On the other hand, both E2Fs and CCDN1 are able to bind the *miR-17-92* promoter establishing a negative feedback loop [32, 51, 52].

The *miR-17* can also act as regulator of cell cycle by directly targeting more than 20 genes involved in the G1/S phase cell cycle transition. In this context, *miR-17*, by inhibiting the mitogen-activated kinase *JNK2*, a cell cycle promoting protein [47], may have also a tumor suppressive role that has been demonstrated by evidence showing that loss of heterozygosity at 13q31.3 is associated with tumor progression and poor prognosis in several cancers [12, 53].

### Interaction of the *miR-17-92* cluster with the B cell receptor (BCR)

The role of the *miR-17-92* cluster has also been investigated in the regulation of the BCR pathway. In this context, information has been obtained by taking advantage of the P493-6 cell line, an Epstein-Barr virus-immortalized lymphoblastoid cell line with a tet-repressible *MYC* gene and a significant enrichment of *MYC* repressed genes, all with the predicted binding sites for miRNAs of the *miR-17-92* cluster [54]. In particular, genes for the ITIM-containing proteins CD22 and CD32b were identified as direct targets of the *miR-17-92* cluster. Moreover, either MYC or *miR-17-92* expression have been found necessary to sustain phosphorylation of the BCR pathway proteins SYK and BLNK upon BCR ligation. Furthermore, stimulation of the BCR response in *miR-17-92* overexpressing cells results in enhanced calcium influx and elevated levels of MYC. Consistently, inhibition of the *miR-17-92* cluster was demonstrated to diminish the BCR response as measured by SYK and BLNK phosphorylation [54].

### Dysregulation of the *miR-17-92* cluster in DLBCL

DLBCL is a heterogeneous lymphoproliferative disease in which different molecular subtypes have been identified involving the deregulation of distinct signalling pathways [55]. Among the three molecular DLBCL subtypes recognized by gene-expression profiling, those classified as germinal centre B cell-like (GCB) DLBCL harbour frequent 13q31.3 amplification. Overexpression of the *miR-17-92* cluster members was a consequence of such an amplification, as found in leukemia/lymphoma cell lines bearing 13q amplification [56], or due to an overexpression of *MYC* [17, 26, 57–59]. In DLBCL, *MYC* gene aberrations were found in less than 10% of the cases at diagnosis [60] and in almost 20% at first relapse [61, 62]. *MYC* aberration in DLBCL usually associates with complex karyotypes and *BCL2* and *BCL6* rearrangements [63]. MYC protein overexpression was recently identified in about 30% of cases, without significant difference between GCB-DLBCL and non-GCB-DLBCL subtypes [62, 64, 65].

Another genetic aberration related to *miR-17-92* cluster overexpression in GCB DLBCL is a complex rearrangement t(3;13)(q27;q31)t(12;13)(p11;q31), which targets both *BCL6* and the *miR-17-92* cluster. In particular, this rearrangement was shown to deregulate *miR-17-92* by increasing histone acetylation at its upstream breakpoint near the *MIR17HG* promoter [56].

DLBCL heterogeneity is also related to differences in anatomical presentation in extranodal sites. An expression profiling study in DLBCL cases with different extranodal locations showed no significant difference in the expression of *miR-17-92* cluster members in comparison with the nodal DLBCLs [66]. Nevertheless, others studies, focused on DLBCL primarily originating from the central nervous system showed higher levels of *miR-20a* and/or *miR-17* compared to nodal DLBCL [66, 67]. Consistently, high levels of *miR-19b-1* and *miR-92a-1* were detected in the cerebrospinal fluid of these lymphomas [68].

In keeping with the capability of miRNAs from the *miR-17-92* cluster to regulate specific BCR pathway genes, DLBCLs characterized by higher levels of many components of the BCR signalling cascade (e.g. CD19, IG, CD79a, BLK, SYK, PLCγ2, and MAP4K) has been found to express higher *MYC* and *miR-17-92* transcript levels than other DLBCL subtypes [54, 69]. In this context, studies investigating copy number variations identified the *MYC* and *MIR17HG* loci as frequently amplified, consistently with the idea that DLBCLs require high levels of MYC or of miRNAs from the *miR-17-92* cluster to sustain BCR response [54, 69].

DLBCL could arise as transformation of follicular lymphoma (FL) or CLL, leading to the so-called Richter's syndrome (RS). In these cases, amplification of the 13q31.3 region containing the *miR-17-92* cluster, described to be acquired at the time of transformation, was coupled with the gain of *MYC* and loss of *TP53* suggesting a late involvement of the *miR-17-92* cluster in the acquisition of a more biologically aggressive phenotype [70, 71].

Finally, individuals infected by HIV have an increased risk for developing non-Hodgkin's lymphomas (AIDS-NHL) including DLBCL, and overexpression of *miR-17-92* and its paralogs have been found in these and other AIDS-NHL subtypes [72].

### Dysregulation of the *miR-17-92* cluster in MCL

Overexpression of the *miR-17-92* cluster in MCL is usually associated with gains and high number amplifications of its locus [24]. The same study evidenced that at least two miRNAs of this cluster, *miR-17* and *miR-20a*, when associated with high *MYC* mRNA levels, identified a MCL subset with a more aggressive clinical behaviour [24]. A correlation of high *miR-17-92* expression with poor prognosis was also confirmed in a subsequent independent study [73]. It has been proposed that this effect is related with the targeting by miRNAs of the *miR-17-92* cluster on several negative regulators of the PI3K/AKT pathway, e.g. *PHLPP2* and *PTEN*, as well as in the blocking of chemotherapy-induced apoptosis through the targeting of *BCL2L11* [19]. Consistently, the knockdown of miRNAs from the *miR-17-92* cluster inhibits tumor growth in xenograft MCL mouse models [19] and the enforced expression of the same miRNAs facilitates cell proliferation and apoptosis suppression of lymphocytes [19, 43]. This cell proliferative effect of miRNAs belonging to this cluster can be also detected in MCL primary samples in studies either involving peripheral blood or lymph node samples [24, 74]. Targeting on additional cell cycle regulators are surely involved in this effect as, for example, it has been shown for *CDKN1A* in MCL cells [75].

### Dysregulation of the *miR-17-92* cluster in BL

In a recent study [76], high *miR-17-92* overexpression has been found in BL, thus confirming that the activation of the MYC*/miR-17-92* axis is a general feature of this disease. In addition, gain of 13q31.3 has been found to occur in about 10–20% of BLs and to associate with *miR-17-92* overexpression [77, 78]. Of note, the *MYC* gene is translocated to one of the immunoglobulin loci in virtually all BLs. In particular, the typical translocation of *MYC* into the immunoglobulin heavy chain locus is observed in about 80% of BLs whereas the variant translocation into either the κ or λ light chain loci occurs at a frequency of about 10% [79]. The juxtaposition of the immunoglobulin heavy chain or of the κ/λ light chain loci with *MYC* are required for the induction of translocated *MYC* transcription [80–82]. The transcription of the translocated *MYC* is greater than that seen in resting B-cells and similar to that of actively dividing non-malignant B cells such those infected by EBV [79]. This overexpression can induce higher *miR-17-92* levels. Moreover, the deletion of *miR-17-92* in established *MYC*-driven lymphoma cell lines decreased the capability to growth in tissue culture and immunodeficient hosts, in keeping with the concept that *miR-17-92* levels influences proliferation of these cells [39].

### Dysregulation of the *miR-17-92* cluster in CLL

In a study in which miRNA expression was compared in CLL with or without *TP53* abnormalities [83], a significant down regulation of *miR-17* in cases harbouring *TP53* deletion/mutation was shown. These data were subsequently confirmed in an another study in which CLL patients lacking *TP53* expression and displaying aggressive disease exhibit reduced *miR-17* and *miR-20a* level of expression and increased *miR-19a*, *miR-19b-1* and *miR-92a-1*, whereas *miR-18a* levels were unchanged, compared to healthy normal controls [44]. In contrast, patients expressing wild type *TP53*, exhibit increased levels of *miR-17* and *miR-20a*, unchanged levels of *miR-18a*, *miR-19a*, *miR-19b-1* and lower levels of *miR-92a-1* [44]. The loss of *TP53* may selectively effect the processing of *miR-17-92* miRNA, resulting in the imbalanced expression of its encoded units [84].

Concerning the ability of miRNA belonging to the *miR-17-92* cluster to predict disease outcome, the debate is still open. In this context, *miR-20a* expression has been found to correlate with time to treatment in CLL [85].

Regarding the capacity of microenvironmental stimuli to induce miRNA expression, in a study of some of us [22], expression of miRNAs from the *miR-17-92* cluster were up-regulated upon TLR9 triggering by CpG, compared to unstimulated cells, in CLL cells bearing an unmutated BCR, whereas no difference was detected in the context of a mutated configuration. In this study, the *miR-17-92* overexpression due to TLR9 stimulation was correlated with the concomitant gene expression signature. In particular, it has been shown that the enforced expression of *miR-17* in primary unmutated *IGHV* CLL cells reduces the expression of the tumor suppressor genes *E2F5*, *TP53INP1*, *TRIM8* and *ZBTB4*. Among these genes, *ZBTB4* and *TP53INP1* are involved in apoptosis regulation through *CDKN1A* and *TP53* [86, 87], *E2F5* is involved in the G1 arrest [88], and *TRIM8*, is involved in the degradation of *SOCS1*, a well-known regulator of the response to CpG [89]. All these genes were identified as direct targets of *miR-17*, as evidenced by their significant downregulation upon ectopic *miR-17* overexpression. In the same study [22], we also demonstrated that the stable and sustained up-regulation of miRNAs belonging to the *miR-17-92* cluster in unmutated *IGHV* CLL by CpG is preceded by induction of *MYC*, thus providing evidence of an associative interaction between *MYC* and *miR-17-92* cluster also in CLL cells. In agreement with these results, *miR-17* transfection was also demonstrated to be sufficient to reduce apoptosis induced by serum deprivation in a series of primary unmutated *IGHV* CLL cells. Consistently, as indicated by experiments in primary CLL cells, transfection with *miR-17-92* cluster antagomiRs reduces bromo-deoxy-uridine incorporation in CpG-stimulated unmutated *IGHV* CLL cells [22]. Moreover, the expression of *miR-17* was demonstrated to be significant higher in unmutated *IGHV* CLL cells expressing high ZAP70 compared with the *IGHV*-mutated/ZAP70-low counterpart [22]. This evidence could suggest that *IGHV*-unmutated/ZAP70-high CLL cells are more frequently subjected to signals capable of activating the TLR9 pathway, or, alternatively, that the former cells have a greater capacity to respond to microenvironmental signals, including those delivered through TLR9 stimulation [90–93].

Further evidence regarding the induction of *miR-17-92* expression by microenvironmental stimuli has been provided in another study in which miRNAs belonging to the *miR-17-92* cluster have been found to be differentially expressed and up regulated by the co-culture with stromal cells with or without the co-stimulation with the CD154 molecule [94]. Specifically, four out of the six members of the *miR-17-92* cluster, i.e. *miR-17*, *miR-20a*, *miR-18a* and *miR-19b-1*, resulted significantly regulated by CD154. Other members of the family were also induced but with lower, not significant extent, i.e. *miR-19a* and *miR-92a-1*. In the same study, it has been also shown that, for all the members of the *miR-17-92* cluster except *miR-92a-1*, stromal cell culture produced an increase in miRNA expression, which was further increased by CD154. In this context, the most highly expressed members of the cluster following CD154 culture were *miR-17* and *miR-19b-1* [94]. Of note, also in this case, *miR-17-92* up-regulation was associated with *MYC*, in particular, *MYC* expression was induced by stromal cell culture with a further increase due to CD154 culture [94].

These data indicate that *miR-17-92* cluster over expression may be not only due to genomic abnormalities but also to microenvironmental stimuli capable to influence miRNA expression [22, 94].

### Future perspectives

The discovery of miRNAs acting as oncogenes or oncosuppressors have introduced novel treatment approaches. The more frequently proposed modality of therapy targeting miRNAs is to silence oncomiRs through anti-miRNAs oligonucleotides or to overexpress oncosuppressors miRNAs using miRNA mimics [95–97]. In general, artificial miRNAs or anti-miRNAs are small, stable, and, theoretically, easy to deliver to cells, possibly by packaging them into lipid based and/or antibody-conjugated nanoparticles [95–97]. However, these oligonucleotides still remain to be fully tested for efficacy and safety for a therapeutic use, although very recently a few phase II clinical trials have been performed [98, 99].

Given the multiple and potent effects on cellular proliferation and apoptosis exerted by the *miR-17-92* cluster, miRNAs belonging to the cluster surely represent attractive targets for cancer therapy. In this context, in a study by us [100], it has been recently demonstrated that *in-vitro* administration of a specific oligonucleotide targeting endogenous *miR-17* effectively reduces *miR-17* expression and the proliferation of CLL-like MEC-1 cells. Consistently, it has been also demonstrated that, when injected *in-vivo* in tumor generated by subcutaneously injected MEC-1 cells in SCID mice, this specific oligonucleotide dramatically reduces tumor growth and significantly increase mouse survival. This evidence could represent a proof of principle for the use of specific anti-miRNAs oligonucleotides targeting miRNAs of the *miR-17-92* cluster as a therapeutic tool in CLL and in lymphoproliferative disorders where *miR-17-92* amplification and/or overexpression have a proved pathogenetic role, as discussed in the present review.
